# Persistence of Delirium in Postacute Care at Skilled Nursing Facilities

**DOI:** 10.1001/jamanetworkopen.2025.0860

**Published:** 2025-03-17

**Authors:** Chan Mi Park, Sandra Shi, Richard N. Jones, Eran D. Metzger, Sarinnapha M. Vasunilashorn, Tamara Fong, Dae Hyun Kim, Sharon K. Inouye

**Affiliations:** 1 Hinda and Arthur Marcus Institute for Aging Research, Hebrew SeniorLife, Boston, Massachusetts; 2 Harvard Medical School, Boston, Massachusetts; 3 Department of Psychiatry and Human Behavior, Warren Alpert Medical School, Brown University, Providence, Rhode Island; 4 Department of Psychiatry, Beth Israel Deaconess Medical Center, Boston, Massachusetts; 5 Department of Medicine, Beth Israel Deaconess Medical Center, Boston, Massachusetts; 6 Department of Epidemiology, Harvard T. H. Chan School of Public Health, Boston, Massachusetts; 7 Department of Neurology, Beth Israel Deaconess Medical Center, Boston, Massachusetts

## Abstract

**Importance:**

The 2014 Improving Medicare Post-Acute Care Transformation (IMPACT) Act aimed to improve postacute care quality and outcomes. This study examines changes in delirium, a key quality indicator, over 5 years following its implementation.

**Objective:**

To compare the persistence and resolution of delirium within skilled nursing facilities (SNFs) between 2014 and 2019.

**Design, Setting, and Participants:**

This cross-sectional study included SNF admissions from a 5% Medicare random sample, with delirium assessments conducted between January 1 and December 31 in 2014 and 2019. The analysis of persistent delirium consisted of patients with delirium at SNF admission and a subsequent delirium assessment during their SNF stay. Analyses were conducted from December 2023 to October 2024.

**Exposures:**

Year 2019 compared with year 2014.

**Main Outcomes and Measures:**

Delirium was measured using the Minimum Data Set (MDS) Confusion Assessment Method at SNF admission and at a subsequent assessment within 30 days of admission. Multinomial logistic regression was used to compare the rates of resolved delirium, persistent delirium, and death between 2014 and 2019 after adjusting for patient characteristics and SNF care factors.

**Results:**

The sample included a total of 432 037 SNF admissions before exclusions and 306 998 after exclusions. For SNF admissions in 2014, 6933 of 162 161 patients (4.3%) had delirium at admission, compared with 3595 of 144 837 patients (2.5%) in 2019. In 2014, there were 6096 patients (mean [SD] age, 80.6 [11.0] years; 3565 women [58.5%]), and in 2019, there were 2778 patients (mean [SD] age, 80.2 [10.7] years; 1546 women [55.7%]) with delirium and follow-up assessments. The adjusted prevalence of persistent delirium decreased from 3347 of 6096 patients (62.3%; 95% CI, 60.2%-64.4%) in 2014 to 1316 of 2778 patients (54.7%; 95% CI, 52.0%-57.4%) in 2019, whereas delirium resolution increased from 1734 of 6096 patients (29.1%; 95% CI, 27.1%-31.1%) in 2014 to 1010 of 2778 patients (37.4%; 95% CI, 34.7%-40.0%) in 2019.

**Conclusions and Relevance:**

This cross-sectional study found a reduction in the prevalence of delirium at SNF admission and an improvement in delirium resolution during the stay in the 5 years following the IMPACT Act. However, the high prevalence of persistent delirium warrants further efforts to improve delirium management in SNF.

## Key Points

**Question:**

Has persistent delirium in skilled nursing facilities (SNFs) decreased since passage of the Improving Medicare Post-Acute Care Transformation (IMPACT) Act?

**Findings:**

In this cross-sectional study of 306 998 Medicare beneficiaries admitted to SNF, delirium prevalence at admission decreased from 4.3% in 2014 to 2.5% in 2019. In those with delirium at SNF admission, persistent delirium decreased from 62.3% to 54.7%, whereas delirium resolution increased from 29.1% to 37.4%.

**Meaning:**

Although improvements in delirium prevalence and resolution were observed among patients admitted to SNFs following the IMPACT Act, the high rate of persistent delirium underscores the need for improved delirium management in SNF.

## Teaser

This cross-sectional study examines the persistence and resolution of delirium among Medicare beneficiaries admitted to skilled nursing facilities between 2014 and 2019.

## Introduction

Annually, more than 2 million Medicare beneficiaries are discharged from acute care hospitals to skilled nursing facilities (SNFs) for postacute care (PAC).^1,2^ Delirium, an acute disorder characterized by fluctuating inattention, confusion, and levels of consciousness,^3,4,5^ is prevalent among older adults in SNFs and is considered an indicator of quality of care.^6,7,8^ Research found that 1 in every 4 patients had delirium upon SNF admission.^4,9^ Alarmingly, 64% of these patients exhibited persistent or worsening symptoms a week later, whereas only 14% fully recovered from delirium.^9^ Because the persistence of delirium in SNFs is associated with poor outcomes, including hospital readmission, mortality, and long-term institutionalization,^4,9,10,11^ identification and management of delirium in this setting hold substantial clinical and policy implications.^12,13,14^

The Confusion Assessment Method (CAM), which was introduced to the Minimum Data Set (MDS) in 2012, is measured for every SNF admission. The introduction of the Improving Medicare Post-Acute Care Transformation (IMPACT) Act of 2014^15,16^ followed by the 2017 updates to the MDS, which added antipsychotic medication reviews and recommended dose reductions, aimed to improve the quality of PAC.^17,18,19^ These initiatives, alongside the implementation of the SNF Value-Based Purchasing Program in 2018, which incentivized facilities to reduce hospital readmissions,^20^ may have facilitated better recognition and management of delirium in SNFs by standardizing assessment and practices, yet their impact has not been previously examined since.^15,16,19^

Thus, we conducted analyses of 2014 and 2019 MDS data to compare rates of persistent delirium, resolved delirium, and mortality during the SNF stay over the 5 years following the implementation of the IMPACT Act. This time frame was selected to provide a sufficient window of opportunity to evaluate the potential outcomes of the policy changes outlined previously. Given that delirium is a common condition with a validated MDS measure, tracking its changes over time presents a unique opportunity to investigate the potential associations of policy interventions with an outcome of substantial clinical relevance.

## Methods

### Data Source and Study Sample

This cross-sectional study was approved by Advarra, which serves as the institutional review board for Hebrew SeniorLife in Boston, Massachusetts. The Advarra institutional review board granted a waiver of informed consent, which is typical for Medicare claims data analysis, because such research is considered minimal risk under 45 CFR §46; however, because the data are not anonymous and publicly available, the study was conducted in accordance with Centers for Medicare & Medicaid Services data use agreements. The study adheres to the Strengthening the Reporting of Observational Studies in Epidemiology (STROBE) reporting guidelines for reporting observational studies.

**Figure 1.**
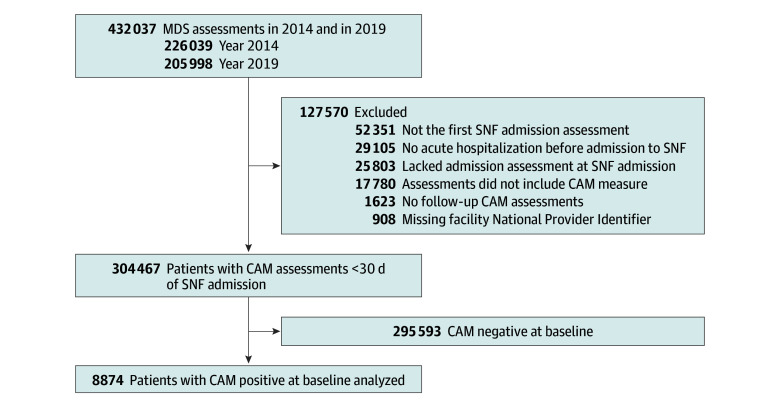
Study Flowchart CAM indicates Confusion Assessment Method; MDS, Minimum Data Set; and SNF, skilled nursing facility.

This study used a nationally representative 5% random sample of Medicare beneficiaries who were admitted to SNF in 2 calendar years: 226 039 SNF admissions in 2014 and 205 998 admissions in 2019. The MDS assessments were from January 1, 2014, to December 31, 2014, and January 1, 2019, to December 31, 2019. The MDS is a federally mandated standardized assessment tool that measures the health status of nursing home residents.^21^ Given that approximately 1.4 million Medicare beneficiaries are admitted to SNFs annually, our sample represents around 4% of the total Medicare beneficiaries each year using SNFs for postacute care.^22,23^ From 432 037 SNF admissions, to avoid duplicate admissions, we included only the first admission per beneficiary in a given year. The exclusion criteria were (1) admissions that were not preceded by an acute hospital discharge to identify postacute SNF admissions, (2) MDS assessment types that do not measure the CAM, (3) missing admission or follow-up assessments, and (4) assessments without a facility National Provider Identifier. Since our focus was on the persistence and resolution of delirium, we further restricted to 8874 admissions in which delirium was present at admission. The details of the selection steps are presented in Figure 1.

### Measurement of Delirium

The primary outcome was the change in delirium status within 30 days of SNF admission. Delirium status was defined by the CAM^3,7^ recorded in the MDS assessment at admission, which is administered within the first 14 days of admission and a subsequent reassessment within 30 days unless the beneficiaries were discharged or died during the SNF stay. The MDS CAM assesses 4 key features: (1) acute onset or fluctuating course, (2) inattention, (3) disorganized thinking, and (4) altered level of consciousness. Delirium was classified as present if the first and second features were present with either the third or fourth features. We focused on beneficiaries with delirium at admission and further classified them into 1 of 3 outcome categories: (1) resolved delirium (ie, delirium at admission but no delirium at the second CAM assessment within 30 days), (2) persistent delirium (ie, delirium at admission and continued delirium at the second CAM assessment within 30 days), and (3) death (ie, delirium at admission but died before a follow-up CAM assessment could be conducted).

### Measurement of Covariates

Demographic information (age, sex, and race), patients’ health conditions, and SNF care–related variables were collected using MDS and Medicare claims data, as described later. Patient race and ethnicity were self-reported in the MDS according to the Office of Management and Budget standards. Race categories included American Indian or Alaska Native, Asian, Black or African American, Native Hawaiian or Other Pacific Islander, and White. Ethnicity was categorized as Hispanic or Latino and not Hispanic or Latino. To comply with Centers for Medicare & Medicaid Services minimum cell size suppression policy, race and ethnicity other than White, Black, and Hispanic were combined into an other category. Data on race and ethnicity were included as covariates to account for potential differences in patient demographics and care practices within SNFs. Health-related variables included Cognitive Function Scale (cognitively intact and mild, moderate, or severe impairment)^24^; activities of daily living dichotomized as independent or dependent; behavioral symptoms that may cause distress to the resident or be disruptive to facility residents, staff members, or the care environment (no behavior, mild to moderate behaviors, or severe to very severe behaviors); responses to the Patient Health Questionnaire–9 (minimal, mild, or moderate to severe depression); hearing and speech (intact or impaired); and the length of PAC stay. We also defined 10 chronic conditions, such as ischemic heart disease, cancer, or dementia, by the Chronic Condition Data Warehouse.^25^ The claims-based frailty index was calculated for a subset of patients who had continuous enrollment in Medicare Parts A and B for 6 months before SNF admission.^26,27^ SNF care–related variables included restraint use, which was recorded as either present or absent, antipsychotic use, and total physical or occupational therapy minutes during the SNF stay. The antipsychotic use was categorized into no use or 1 day or longer.

### Statistical Analysis

Analyses were conducted from December 2023 to October 2024. Multiple imputations by chained equations were used to address missing data, which ranged from 0.03% for the activities of daily living variable to 7.8% for Cognitive Function Scale, to minimize potential biases due to informative missingness. The imputation model included 16 covariates, and 10 imputed datasets were created. After imputation, we compared the characteristics of the study population between 2014 and 2019 using *t* test and χ^2^ test in one of the imputed datasets.

The outcome analysis was conducted using multinomial logistic regression to calculate relative risk ratios (RRRs), comparing the probabilities of persistent delirium or death to resolved delirium (reference outcome) between 2014 and 2019, while accounting for within-SNF outcome correlations using robust variance. We sequentially included different sets of covariates in the model: (1) patient demographic characteristics, (2) patient health–related variables, and (3) SNF care–related variables. Stratified analyses by age, sex, dementia, and frailty status were also conducted to evaluate the associations within each of these groups. All analyses were performed using Stata statistical software version 18.0 (StataCorp). A 2-sided *P *< .05 was considered statistically significant.

In a sensitivity analysis, to assess potential bias from restricting our population to those with both baseline and follow-up CAM assessments, we expanded our cohort to include patients with missing follow-up CAM assessments (1623 patients [15%]). Characteristics of beneficiaries with and without a follow-up CAM assessment are presented in eTable 1 in Supplement 1. We used multiple imputations to assign values to missing follow-up CAM assessments—that is, imputing delirium status for the second (within 1 month) CAM assessment. The same outcome analysis models used previously were repeated, and the results were compared with the primary analyses. All outcome analyses used pooled estimates by combining estimates from 10 imputed datasets.

## Results

### Prevalence of Delirium at SNF Admission

Of 162 161 beneficiaries admitted to SNFs in 2014, 6933 (4.3%) had delirium at admission. In 2019, 3595 of 144 837 patients (2.5%) had delirium at admission. For the subsequent analysis, we restricted the cohort to patients with delirium at admission who had follow-up CAM assessments to evaluate our primary outcome (8874 patients).

### Patient Characteristics and SNF Care–Related Variables

#### Patient Characteristics

Among 6096 patients with delirium at admission to SNF in 2014 and 2778 patients admitted in 2019, the mean age (SD) was similar between the 2 groups (80.6 [11.0] years in 2014 vs 80.2 [10.7] years in 2019) (Table 1). The proportion of female patients decreased slightly from 3565 (58.5%) in 2014 to 1546 (55.7%) in 2019. Racial distributions were comparable: there were 603 Black patients (9.9%) in 2014 and 290 Black patients (10.4%) in 2019, 211 Hispanic patients (3.5%) in 2014 and 79 Hispanic patients (2.8%) in 2019, 4985 White patients (81.8%) in 2014 and 2269 White patients (81.7%) in 2019, and 297 patients in the other category (4.9%) in 2014 and 140 patients in the other category (5.0%) in 2019. Compared with those who were admitted in 2014, patients admitted in 2019 were less likely to have severe cognitive impairment (975 patients [16.0%] vs 374 patients [13.5%]), speech impairment (1525 patients [25.0%] vs 586 patients [21.1%]), and hearing impairment (2011 patients [33.0%] vs 856 patients [30.8%]) (Table 1).

**Table 1.** Patient Characteristics and Skilled Nursing Facility Care–Related Variables for Patients With Delirium in 2014 and 2019^a^

Characteristics	Patients, No. (%)		*P* value
	2014 (n = 6096)	2019 (n = 2778)	
Patient characteristics			
Age, mean (SD), y	80.6 (11.0)	80.2 (10.7)	.07
Sex			
Female	3565 (58.5)	1546 (55.7)	.01
Male	2531 (41.5)	1232 (44.4)	
Race and ethnicity			
Black	603 (9.9)	290 (10.4)	.41
Hispanic	211 (3.5)	79 (2.8)	
White	4985 (81.8)	2269 (81.7)	
Other^b^	297 (4.9)	140 (5.0)	
Cognitive Function Scale score			
Cognitively intact	491 (8.1)	223 (8.0)	.01
Mild impairment	1151 (18.9)	540 (19.4)	
Moderate impairment	3479 (57.1)	1641 (59.1)	
Severe impairment	975 (16.0)	374 (13.5)	
Hearing impairment	2011 (33.0)	856 (30.8)	.01
Speech impairment	1525 (25.0)	586 (21.1)	<.001
Activities of daily living dependency			
Bed mobility	5788 (95.0)	2618 (92.2)	.04
Personal hygiene	5836 (95.7)	2625 (94.5)	.04
Dressing	5959 (97.8)	2698 (97.1)	.19
Bathing	6011 (98.6)	2717 (97.8)	.02
Toileting	5936 (97.4)	2683 (96.6)	.04
Eating	3378 (55.4)	1315 (47.3)	<.001
Total activities of daily living dependence score, mean (SD)	7.28 (1.20)	7.13 (1.34)	<.001
Comorbidity			
Diabetes	2833 (46.5)	1288 (46.4)	.92
Atrial fibrillation	1743 (28.6)	711 (25.6)	.003
Heart failure	3179 (52.2)	1278 (46.0)	<.001
Ischemic heart disease	3985 (65.4)	1736 (62.5)	.009
Chronic obstructive pulmonary disease	2525 (41.1)	1057 (38.1)	.003
Osteoporosis	1876 (30.8)	782 (28.2)	.01
Chronic kidney disease	3125 (51.3)	1613 (58.1)	<.001
Hip fracture history	1081 (17.7)	457 (16.5)	.14
Cancer	1188 (19.5)	531 (19.1)	.68
Dementia	4430 (64.2)	2281 (63.5)	.49
Behaviors (physical and verbal aggression)			
No behaviors	4090 (67.1)	1903 (68.5)	<.001
Mild to moderate behaviors	1241 (20.4)	604 (21.7)	
Severe to very severe behaviors	765 (12.6)	271 (9.8)	
Patient Health Questionnaire–9			
Minimal depression	3307 (54.3)	1613 (58.1)	<.001
Mild depression	1349 (22.1)	513 (18.5)	
Moderate to severe depression	1440 (23.6)	652 (23.5)	
Skilled nursing facility care–related variables			
Antipsychotic use in the past week			
Not used	4345 (71.3)	2028 (73.0)	.23
≥1 d	1751 (28.7)	750 (27.0)	
Use of restraints in the past week	189 (3.1)	29 (1.0)	<.001
Occupational therapy duration, mean (SD), min	207.5 (108.8)	204.2 (109.2)	.19
Physical therapy duration, mean (SD), min	217.5 (114.2)	210.5 (120.1)	.009
Length of stay, mean (SD), d	19.0 (9.0)	17.8 (8.8)	<.001

^a^ Characteristics are obtained from one of the imputed datasets.

^b^ Other includes American Indian or Alaska Native, Asian, and, Native Hawaiian or Other Pacific Islander.

#### SNF Care–Related Variables

Although there was a slight change in antipsychotic use, it was not statistically significant, with 750 patients (27.0%) using antipsychotics more than 1 day in 2019 compared with 1751 patients (28.7%) in 2014 (Table 1). Patients in 2019 had significantly lower use of restraints (189 patients [3.1%] vs 29 patients [1.0%]), shorter lengths of stay (mean [SD], 19.0 [9.0] days vs 17.8 [8.8] days), and reduced time in occupational therapy (mean [SD], 207.5 [108.8] minutes vs 204.2 [109.2] minutes) and physical therapy (mean [SD], 217.5 [114.2] minutes vs 210.5 [120.1] minutes) (Table 1).

This likely explains their earlier discharge from SNFs, resulting in the absence of follow-up CAM assessments within the 30-day window. This observation highlights the necessity for a sensitivity analysis, including patients without follow-up CAM assessments.

### Changes in Delirium Outcomes

**Figure 2.**
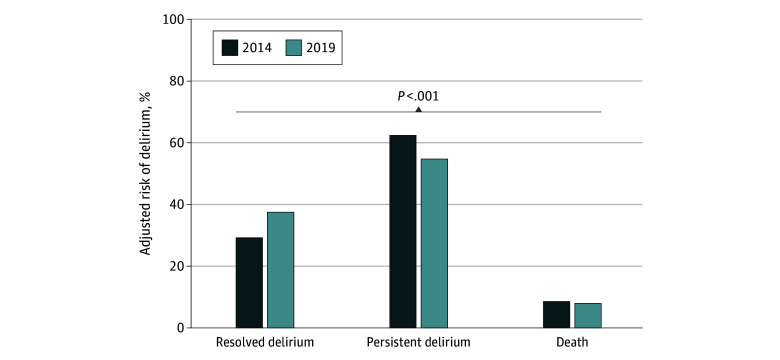
Comparison of Outcomes Between 2014 and 2019 Bar graph shows the adjusted risk of delirium status in skilled nursing facilities, comparing data from 2014 and 2019. The proportion of resolved delirium increased from 29.1% to 37.4%, whereas the proportion of persistent delirium decreased from 62.3% to 54.7%.

Among patients who had delirium at SNF admission, there was a substantial increase in the frequency of delirium resolution, from 1734 of 6096 patients (29.1%; 95% CI, 27.1%-31.1%) in 2014 to 1010 of 2778 patients (37.4%; 95% CI, 34.7%-40.0%) in 2019; a decrease in persistent delirium, from 3347 of 6096 patients (62.3%; 95% CI, 60.2%-64.4%) in 2014 to 1316 of 2778 patients (54.7%; 95% CI, 52.0%-57.4%) in 2019; and a decrease in mortality within 30 days, from 1015 of 6096 patients (8.6%; 95% CI, 7.4%-9.8%) in 2014 to 452 of 2778 patients (7.9%; 95% CI, 6.7%-9.3%) in 2019, after adjusting for demographic, health conditions, and SNF care factors (Figure 2). The multinomial logistic regression analysis demonstrated a reduction in the risk of persistent delirium vs resolved delirium in 2019 compared with 2014, with an RRR of 0.68 (95% CI, 0.61-0.76) (Table 2). Sequential adjustment for patient demographics, health-related variables, and SNF care–related factors did not change the RRR estimates appreciably. Similarly, the risk of death vs resolved delirium within 30 days was significantly lower in 2019, with the RRR improving from 0.76 (95% CI, 0.67-0.88) in the unadjusted model to 0.72 (95% CI, 0.62-0.85) in the fully adjusted model (Table 2). All subgroups defined by age, sex, dementia status, and frailty levels demonstrated a consistent results, showing a significant reduction of persistent delirium in 2019 compared with 2014, particularly among the female, frail, and dementia subgroups (Table 3).

**Table 2.** Comparison of Rates of Persistent Delirium and Mortality Between 2014 and 2019

Outcomes (n = 8874)	Events, No. (%)^a^		Relative risk ratio (95% CI), 2019 vs 2014			
	2014 (n = 6096)	2019 (n = 2778)	Model 1: unadjusted	Model 2: model 1 plus demographic factors	Model 3: model 2 plus health-related conditions^b^	Model 4: model 3 plus SNF care–related factors^c^
Resolved	1734 (28.4)	1010 (36.4)	1 [Reference]	1 [Reference]	1 [Reference]	1 [Reference]
Persistent delirium	3347 (54.9)	1316 (47.4)	0.68 (0.61-0.75)	0.68 (0.61-0.75)	0.68 (0.61-0.76)	0.68 (0.61-0.76)
Death within 30 d	1015 (16.7)	452 (16.3)	0.76 (0.67-0.88)	0.76 (0.66-0.88)	0.80 (0.69-0.92)	0.72 (0.62-0.85)

Abbreviation: SNF, skilled nursing facility.

^a^ Event numbers are obtained from one of the imputed datasets.

^b^ Health-related conditions include 11 chronic conditions, cognitive function scale, patient depression scale, behavioral symptoms.

^c^ SNF care–related conditions include antipsychotic use days, restraint use, total occupational or physical therapy minutes.

**Table 3.** Comparison of Rates of Persistent Delirium and Mortality Between 2014 and 2019 Stratified by Age, Sex, Frailty, and Dementia Status After Multivariable Adjustment

Subgroups	Outcome: persistent delirium		Outcome: death within 30 d	
	2019 vs 2014, RRR (95% CI)	*P* value	2019 vs 2014, RRR (95% CI)	*P* value
Age, y				
<80 (n = 3554)	0.69 (0.58-0.81)	<.001	0.65 (0.50-0.83)	.001
≥80 (n = 5320)	0.68 (0.59-0.78)	<.001	0.78 (0.63-0.96)	.02
Sex				
Male (n = 3763)	0.70 (0.60-0.83)	<.001	0.83 (0.66-1.04)	.12
Female (n = 5111)	0.66 (0.58-0.77)	<.001	0.64 (0.51-0.79)	.51
Frailty				
Nonfrail^a^ (n = 1617)	0.78 (0.61-1.00)	.05	1.01 (0.67-1.54)	.96
Frail^a^ (n = 3664)	0.64 (0.54-0.75)	<.001	0.85 (0.65-1.11)	.25
Dementia				
No dementia (n = 3097)	0.70 (0.58-0.84)	<.001	0.73 (0.57-0.93)	.01
Dementia (n = 5777)	0.68 (0.59-0.77)	<.001	0.72 (0.59-0.89)	.002

Abbreviation: RRR, relative risk ratio.

^a^ Frailty subgroup analyses were conducted on patients for whom the claims-based frailty index was calculated based on continuous enrollment in Medicare Parts A and B for 6 months prior to skilled nursing facility admission.

### Sensitivity Analysis

We observed that individuals who had the CAM measured only at baseline tended to have shorter lengths of stay and were generally healthier compared with those assessed at both baseline and follow-up (eTable 1 in Supplement 1). These patients also exhibited lower rates of severe cognitive impairments and comorbidities, suggesting they had less complex health needs. Including 1623 patients with missing follow-up CAM assessments using multiple imputation (eTable 2 in Supplement 1), we found a significant reduction in the risk of persistent delirium in 2019 compared with 2014, with an RRR of 0.70 (95% CI, 0.62-0.79) in the fully adjusted model. Similarly, the risk of death within 30 days was also significantly lower in 2019, with an RRR of 0.58 (95% CI, 0.50-0.68). These findings confirmed the consistency of the results and supported the robustness of our primary analysis findings.

## Discussion

Our cross-sectional study highlights notable improvements in delirium resolution rates among patients admitted to SNF for PAC from 2014 to 2019 (from 29.1% to 37.4%), accompanied by a reduction in both persistent delirium (from 62.3% to 54.7%) and death (from 8.6% to 7.9%) within 30 days of SNF stay. Despite the general improvement in health characteristics among patients admitted to SNFs in 2019 compared with those admitted in 2014, these improvements were not attributable to differences in demographic characteristics, health-related conditions, and SNF care–related factors. Nevertheless, a substantial proportion of patients, approximately 50%, continued to have delirium during their SNF stay.

Several factors may have contributed to the improvements in delirium management in SNFs between 2014 and 2019. Although the direct association of the policy changes with this improvement cannot be determined from our analyses, the alignment of these positive trends with the enactment of the IMPACT Act in 2014 and enhancements to the MDS assessments, including antipsychotic medication reviews in 2017, suggests at least an indirect influence of these initiatives. Moreover, our results parallel those of previous studies^28,29,30^ that have indicated other improvements associated with the enactment of the IMPACT Act in 2014, such as enhanced care coordination and quality reporting. The standardization of patient assessments has facilitated more accurate identification of patient needs and more consistent management practices, contributing to improved patient outcomes.^31,32^ In addition, the 2017 updates of the MDS assessments specifically targeted the use of off-label antipsychotic medications, with more in-depth assessments aimed at reducing their use whenever possible.^33,34^ Off-label use of antipsychotics has been common in managing delirium^35,36,37^ and has been associated with adverse outcomes.

The decrease in the prevalence of delirium at the time of SNF admission (4.3% in 2014 vs 2.5% in 2019) may also reflect the efforts of acute hospitals to prevent and manage delirium. The decrease may reflect potential benefits from interventions such as the Hospital Elder Life Program, which has resulted in models, protocols, pathways, and guidelines implemented around the world.^38,39,40^ More recently, it has led to the Age-Friendly Health Systems initiative,^41^ which implements the 4Ms framework (What Matters, Medication, Mentation, and Mobility). It has likely contributed to increased awareness and implementation of delirium prevention protocols in acute hospital settings.^41,42^ Proactive nonpharmacological interventions in acute hospital settings can reduce the severity and duration of delirium,^5,43^ facilitating smoother transitions to PAC.^44^

The strengths of this study include the systematic evaluation of delirium outcomes in PAC, an understudied care setting. The study is novel and important in its use of nationally representative MDS data to examine changes in delirium status (ie, persistence and resolution of delirium and mortality) in the SNF setting related to Centers for Medicare & Medicaid Services policy interventions in 2014. The use of multiple imputations and consistent results across multiple subgroups further strengthen our findings.

### Limitations

This study has limitations that should be mentioned. First, CAM assessment recorded in MDS may be less accurate than research assessment. Kiely et al^44^ reported a delirium rate of 16% in SNFs with research assessments, higher than the 4.3% observed in our study, suggesting the possibility of underdetection of delirium within the MDS assessment. However, our findings align with a 2017 national study reporting a delirium incidence of 4.3% during postacute SNF admissions, consistent with our 2014 rate.^14^ Second, 15% of patients were missing the follow-up CAM assessment. These individuals might be healthier compared with those with both baseline and follow-up assessments, which may explain a shorter length of stay and the lack of a follow-up CAM assessment. Third, facility-level factors (eg, bed size or staff-to-patient ratios) that may influence delirium outcomes were not available in the MDS dataset. To address this limitation, we accounted for facility-level variability using cluster analyses by National Provider Identifier. Moreover, additional policy changes during the study period, such as the Patient-Driven Payment Model (2019), may have influenced delirium reporting or management practices, adding complexity to the interpretation of results. Furthermore, this study involves separate cross-sections of patients in 2014 and 2019; thus, we could not track individual changes over time, and the results may also be subject to temporal changes.

## Conclusions

This cross-sectional study demonstrates improvements in the resolution of delirium among patients admitted to SNFs between 2014 and 2019, with reductions in both persistent delirium and 30-day mortality. However, the rate of persistent delirium remains concerningly high. Given the negative health consequences of persistent delirium,^11,12,13,14,45^ our findings call for delirium prevention and management strategies in the SNF PAC setting, such as staff training for identifying delirium, the adoption of a systematic delirium prevention and management program such as Hospital Elder Life Program, and multidisciplinary strategies to improve patient care in SNFs.

## Notes

Supplement 1. **eTable 1.** Characteristics of Patients with Delirium upon Admission to SNF: A Comparative Analysis of 2014 and 2019 Data with and without Second CAM Measure **eTable 2.** Sensitivity Analysis: Comparison of Rates of Persistent Delirium and Mortality Between 2014 and 2019 by Including Patients without Follow-up CAM using Multiple Imputation

Supplement 2.
Data Sharing Statement
