# Supplementary material for: Persistence of Delirium in Postacute Care at Skilled Nursing Facilities
Source: JAMA Netw Open. 2025 Mar 17;8(3):e250860. doi: 10.1001/jamanetworkopen.2025.0860 (PMC11915067; doi:10.1001/jamanetworkopen.2025.0860)
Supplement: Supplement 1. — eTable 1. Characteristics of Patients with Delirium upon Admission to SNF: A Comparative Analysis of 2014 and 2019 Data with and without Second CAM Measure eTable 2. Sensitivity Analysis: Comparison of Rates of Persistent Delirium and Mortality Between 2014 and 2019 by Including Patients without Follow-up CAM using Multiple Imputation [file jamanetwopen-e250860-s001.pdf]

Supplemental Online Content

Park CM, Shi S, Jones RN, et al. Persistence of delirium in postacute care at skilled nursing facilities. *JAMA Netw Open*. 2025;8(3):e250860. doi:10.1001/jamanetworkopen.2025.0860

eTable 1. Characteristics of Patients with Delirium upon Admission to SNF: A Comparative Analysis of 2014 and 2019 Data with and without Second CAM Measure
eTable 2. Sensitivity Analysis: Comparison of Rates of Persistent Delirium and Mortality Between 2014 and 2019 by Including Patients without Follow-up CAM using Multiple Imputation

This supplemental material has been provided by the authors to give readers additional information about their work.

© 2025 Park CM et al. *JAMA Network Open*.

eTable 1. Characteristics of Patients with Delirium upon Admission to SNF:
A Comparative Analysis of 2014 and 2019 Data with and without Second CAM Measure

Characteristics, n (%)	2014		2019	
	CAM measured at baseline and follow-up within 30 days (n=6,096)	CAM measured only at baseline (n=808)	CAM measured at baseline and follow-up within 30 days (n=2,778)	CAM measured only at baseline (n=815)
**Age, mean (SD)**	80.6 (11.0)	76.7 (12.5)	80.2 (10.7)	78.8 (11.3)
**Female**	3,578 (58.4)	459 (58.7)	1,555 (55.8)	487 (60.5)
**Race**				
White	5,002 (81.7)	609 (77.9)	2,276 (81.6)	663 (82.4)
Black	610 (10.0)	96 (12.3)	293 (10.5)	73 (9.1)
Hispanic	211 (3.5)	32 (4.1)	79 (2.8)	26 (3.2)
Other	299 (4.9)	45 (5.8)	140 (5.0)	43 (5.3)
**CFS Scale**				
Cognitively Intact	462 (7.6)	68 (8.7)	211 (7.6)	62 (7.7)
Mild Impairment	1,076 (17.6)	138 (17.7)	515 (18.5)	146 (18.1)
Moderate Impairment	3,243 (53.0)	389 (49.7)	1,532 (55.0)	439 (54.5)
Severe Impairment	875 (14.3)	101 (12.9)	331 (11.9)	92 (11.4)
CFS missing	466 (7.6)	86 (11.0)	199 (7.1)	66 (8.2)
**Hearing impairment**	1,997 (32.6)	204 (26.1)	855 (30.7)	209 (26.0)
Hearing missing	62 (1.0)	11 (1.4)	23 (0.8)	NA
**Speech impairment**	1,521 (24.8)	171 (21.9)	583 (20.9)	168 (20.9)
Speech missing	35 (0.6)	NA	18 (0.7)	NA
**ADL dependency**				
Bed mobility	5,812 (94.9)	678 (86.7)	2,628 (94.3)	706 (87.7)
Personal hygiene	5,861 (95.7)	692 (88.5)	2,635 (94.5)	742 (92.2)
Dressing	5,984 (97.8)	723 (92.5)	2,708 (97.1)	752 (93.4)

© 2025 Park CM et al. *JAMA Network Open*.

Bathing	6,037 (98.6)	741 (94.8)	2,727 (97.8)	782 (97.1)
Toileting	5,961 (97.4)	709 (90.7)	2,693 (96.6)	744 (92.4)
Eating	3,395 (55.5)	336 (43.0)	1,320 (47.4)	328 (40.8)
Total ADL dependence score, mean (SD)	7.3 (1.2)	6.7 (2.0)	7.1 (1.3)	6.8 (1.8)
**Comorbidity**				
Diabetes	2,847 (46.5)	318 (40.7)	1,292 (46.3)	343 (42.6)
Atrial fibrillation	1,751 (28.6)	142 (18.2)	713 (25.6)	158 (19.6)
Heart failure	3,191 (52.1)	327 (41.8)	1,284 (46.1)	298 (37.0)
Ischemic heart disease	4,004 (65.4)	430 (55.0)	1,743 (62.5)	427 (53.0)
COPD	2,538 (41.5)	299 (38.2)	1,061 (38.1)	266 (33.0)
Stroke	2,267 (37.0)	232 (29.7)	977 (35.0)	229 (28.5)
Osteoporosis	1,887 (30.8)	188 (24.0)	785 (28.2)	204 (25.3)
CKD	3,142 (51.3)	319 (40.8)	1,619 (58.1)	414 (51.4)
Hip fracture history	1,089 (17.8)	95 (12.2)	461 (16.5)	109 (13.5)
Cancer	1,195 (19.5)	113 (14.5)	532 (19.1)	117 (14.5)
Dementia	4,007 (65.5)	423 (54.1)	1,793 (64.3)	488 (60.6)
**Behaviors**				
No behaviors	4,030 (65.8)	474 (60.6)	1,900 (68.2)	491 (61.0)
Mild to moderate behaviors	1,229 (20.1)	177 (22.6)	594 (21.3)	204 (25.3)
Severe to very severe behaviors	729 (11.9)	114 (14.6)	266 (9.5)	98 (12.2)
Missing	134 (2.2)	17 (2.2)	28 (1.0)	12 (1.5)
**PHQ-9**				
Minimal depression	3,236 (52.9)	408 (52.2)	1,588 (57.0)	445 (55.3)
Mild depression	1,310 (21.4)	166 (21.2)	498 (17.9)	148 (18.4)
Moderate to severe depression	1,400 (22.9)	162 (20.7)	630 (22.6)	187 (23.2)
Missing	176 (2.9)	46 (5.9)	72 (2.6)	25 (3.1)

© 2025 Park CM et al. *JAMA Network Open*.

**Antipsychotic Use in the past week**				
Not used	4,360 (71.2)	543 (69.4)	2,035 (73.0)	535 (66.5)
1-5 days	450 (7.4)	79 (10.1)	193 (6.9)	101 (12.6)
≥6 days	1,035 (21.3)	155 (19.8)	559 (20.1)	169 (21.0)
**Use of restraints in the past week**	189 (3.1)	23 (2.9)	29 (1.0)	NA
**Occupational therapy minutes, mean (SD)**	207.6 (108.7)	145.7 (114.1)	204.4 (109.1)	142.2 (102.9)
**Physical therapy minutes, mean (SD)**	217.6 (114.1)	155.9 (118.4)	210.7 (119.9)	147.5 (107.6)
**Length of stay, mean (SD)**	19.0 (9.0)	6.7 (4.6)	17.8 (8.8)	6.7 (4.2)

© 2025 Park CM et al. *JAMA Network Open*.

eTable 2. Sensitivity Analysis: Comparison of Rates of Persistent Delirium and Mortality Between 2014 and 2019 by Including Patients without Follow-up CAM using Multiple Imputation

Models (N=10,497)	Outcome: Persistent Delirium		Outcome: Death within 30 days	
	2019 vs 2014 RRR (95% CI)	p-value	2019 vs 2014 RRR (95% CI)	p-value
Model 1: Unadjusted	0.71 (0.62-0.79)	<0.01	0.67 (0.58-0.77)	<0.01
Model 2: Model 1 + demographic factors	0.70 (0.62-0.79)	<0.01	0.67 (0.58-0.77)	<0.01
Model 3: Model 2 + health-related conditions[a]	0.70 (0.63-0.79)	<0.01	0.69 (0.59-0.79)	<0.01
Model 4: Model 3 + SNF care-related factors[b]	0.70 (0.62-0.78)	<0.01	0.58 (0.50-0.68)	<0.01

Abbreviations: CI, confidence interval; RRR, relative risk ratio; SNF, skilled nursing facility
[a]Health-related conditions include 11 chronic conditions, cognitive function scale, patient depression scale, behavioral symptoms
[b]SNF care-related conditions include antipsychotic use days, restraint use, total occupational or physical therapy minutes

© 2025 Park CM et al. *JAMA Network Open*.
