# Supplementary material for: Persistence of Delirium in Postacute Care at Skilled Nursing Facilities
Source: JAMA Netw Open. 2025 Mar 17;8(3):e250860. doi: 10.1001/jamanetworkopen.2025.0860 (PMC11915067; doi:10.1001/jamanetworkopen.2025.0860)
Supplement: Supplement 2. — Data Sharing Statement [file jamanetwopen-e250860-s002.pdf]

Data Sharing Statement

Park. Persistence of Delirium in Postacute Care at Skilled Nursing Facilities. *JAMA Netw Open*. Published March 17, 2025. doi:10.1001/jamanetworkopen.2025.0860

Data
Data available: No

Additional Information
Explanation for why data not available: We are unable to share data due to CMS data privacy rules. Medicare claims data are available through data use agreement with CMS.
